# The influence of socioenvironmental risk factors on risk-taking behaviors among Bahamian adolescents: a structural equation modeling analysis

**DOI:** 10.1080/21642850.2023.2297577

**Published:** 2024-01-08

**Authors:** Elizabeth Schieber, Ava Wang, Grace Ou, Carly Herbert, Hoa T. Nguyen, Lynette Deveaux, Xiaoming Li

**Affiliations:** a Department of Population and Quantitative Health Sciences, UMass Chan Medical School, Worcester, MA, USA; b High School Internship Program with UMass Chan, Lexington High School, Lexington, MA, USA; c Office of HIV/AIDs, Ministry of Health, Nassau, The Bahamas; d Department of Health Promotion, Education, and Behavior, University of South Carolina Arnold School of Public Health, Columbia, SC, USA

**Keywords:** Parental monitoring, adolescent risk behavior, environmental risk, Bahamas, structural equation modeling

## Abstract

**Background:**

Adolescents’ risk-taking behaviors can have profound impacts on their future health. Few studies have established a relationship between multiple social environmental factors and adolescent risk behaviors. We used structural equation modeling to examine the role of parental monitoring and environmental risks on adolescents’ behavioral intentions and risk behaviors.

**Methods:**

Data were collected through the baseline survey of a national implementation project among 2205 Grade 6 students in 24 government schools in The Bahamas in 2019. Structural equation modeling examined relations among parental monitoring, environmental risk factors, behavioral intentions, and risk behaviors.

**Results:**

Students had engaged in various delinquent, substance use, and sexual risks. In the structural equation model, parental monitoring demonstrated direct negative (protective) effects on behavioral intentions and risk behaviors, whereas environmental risk factors had a direct positive effect on adolescent behavioral intentions and risk behaviors. The model had an R^2^ value of 0.57 for adolescent risk behaviors.

**Conclusion:**

Parental monitoring and environmental risk factors had strong influences on risk-taking behaviors of early adolescents. Future adolescent health behavior interventions should consider offering additional prevention resources to early adolescents who are exposed to multiple environmental risk factors.

Adolescence is a vulnerable developmental stage when physical and cognitive maturation can lead to heightened risk-taking and experimentation (Willoughby et al., 2021). The interaction of social, environmental, and personal factors during adolescence forms individuals’ foundations for health-related decision-making (Smith et al., 2008). Experimentation is likely during this developmental stage as adolescents assert their independence and autonomy (Duell & Steinberg, 2019). Risk-taking typically emerges during early adolescence (around ages 10-12) and follows an inverted U pattern where risk-taking increases then decreases with age into adulthood (Duell et al., 2018; Romer et al., 2017), making early adolescence an important developmental period to capture behavioral risk factors. Although some may establish positive habits through experimentation, many adolescents participate in risky behaviors such as substance use, delinquent behaviors, and sexual risk behaviors. These risk behaviors can result in negative downstream health consequences, including sexually transmitted infections and HIV, unintended pregnancies (Kotchick et al., 2001; Ningpuanyeh, 2015), substance use disorders (Spring et al., 2012), and premature death (Huesmann et al., 2009; Spring et al., 2012). Negative consequences of adolescent risk behaviors can go beyond physical health. Adolescents with high levels of risk behaviors increase their likelihood of leaving school prematurely (Mohr et al., 2019), are more likely to be a victim of or perpetuate crime (Carter, 2019), and can experience employment challenges (Carter, 2019). Thus, mitigating adolescent risk behaviors can increase individuals’ long-term health and well-being.

## Developmental ecological systems framework of risk behaviors

The social-ecological model examines the interplay among intrapersonal-, interpersonal-, community-, and societal-level factors that influence peoples’ health (Dahlberg, 2002). This framework is used to describe the social factors that shape individuals’ development and behavior over time. Previous literature supports the individual association of each factor with risk-taking behaviors; however, it is unclear how these community, interpersonal, societal, and intrapersonal factors interact. We looked at individuals’ age, sex, and behavioral intentions at the intrapersonal level, parental monitoring at the interpersonal level, and neighborhood/environmental risk factors at the community level.

### Intrapersonal factors

Age and gender are two significant demographic characteristics that are related to adolescent risk taking. Older adolescents are typically afforded more independence and opportunities to experiment with risky behaviors (Duell & Steinberg, 2019). Cultural expectations may differ between genders, affording differential opportunities to participate in risky behaviors (Dávila et al., 2017). For example, Dávila et al. (2017) observed that girls in Mexico received more parental supervision than boys, highlighting some of the differences between how boys and girls were treated in that culture. Age and puberty also have effects on adolescent brain development, and early initiation of substance use can affect male and female brain development differently (Blakemore et al., 2010). For example, female adolescents with high levels of alcohol use are more likely to have reduced prefrontal cortex volumes while males have larger volumes than average, indicating that females may become more impaired by alcohol (Bava & Tapert, 2010). Testosterone levels have been linked to increased alcohol intake (Vijayakumar et al., 2018), thus when testosterone levels increase during puberty, males may be more likely to consume more alcohol. These biological and social factors related to age and gender interact with other environmental factors to shape adolescents’ development, in a critical development period for establishing healthy habits.

Individuals’ behavioral intentions i.e. their motivations or attitudes toward participating in a particular behavior, lead to engagement in that behavior (Ajzen, 1991). A meta-analysis of behavior-change interventions found that medium-to-large changes in behavior intentions predicted small-to-medium changes in behaviors (Webb & Sheeran, 2006). Age and inherent maturation also play a significant role in determining whether one acts on their intentions (Duell et al., 2018). Development of the adolescent brain contributes to impulsivity. While behavior intentions are not perfect predictors of future behavior, they are still important mediators of behavior, and studying factors that can influence intentions is important for identifying at-risk adolescents and potential avenues for intervention.

### Interpersonal factors

Parents significantly influence adolescents’ behavior (Jones et al., 2012; Wang et al., 2013; Yu et al., 2006) Previous studies have demonstrated a consistent relationship between higher levels of parental monitoring and lower levels of problem behaviors, including delinquency (Wang et al., 2015), substance use (Barnes et al., 2006), and sexual risk behaviors (Dávila et al., 2017; Huang et al., 2012). Although parental monitoring has been found to reduce a range of risky behaviors (DeVore & Ginsburg, 2005), few studies have simultaneously examined how parental monitoring interacts with other environmental factors to influence adolescent risk behaviors, and those that have examined these important relations between multilevel factors have largely been focused in developed, Western countries such as the US (Chuang et al., 2005; Gardner et al., 2012; Orihuela et al., 2020) Positive parental relationships have been shown to mitigate neighborhood risk factors for early sexual initiation (Gardner et al., 2012; Orihuela et al., 2020), substance use (Chuang et al., 2005), and recidivism of troubled youth (Voisin et al., 2012).

### Community factors

Neighborhood/environmental risk factors, such as the presence of violence, substance use, and peers with high levels of risk behaviors, also affect adolescents’ risk behaviors (Browning et al., 2015; Kerr et al., 2015). Socioeconomic disadvantaged neighborhoods are associated with a multitude of adolescents’ problem behaviors such as conduct problems, violence, and sexual risk behaviors (Chang et al., 2016; McBride Murry et al., 2011). Further, neighborhoods with stronger social networks between households and peer groups that exhibit prosocial behaviors can act as a protective factor against adolescent risk behaviors (Browning et al., 2015). The interplay of the various ecological systems shapes individuals’ learning histories and development, which can in turn shape their behavioral intentions and later participation in risky behaviors.

## Current study

Most studies analyze risk behaviors independently of one another; however, those who engage in one risk behavior are likely to engage in others (Donovan & Jessor, 2016). For example, adolescent substance use (tobacco, alcohol, or drug use) has been found to be associated with sexual risk behaviors such as unprotected sex and having multiple sexual partners (Yan et al., 2007). In this analysis, we simultaneously examined three types of adolescent risk behaviors, delinquency, substance use, and sexual risks, and how environmental risk factors and parental monitoring affected adolescents’ risk behaviors. Caribbean adolescents are an understudied population that engages in high levels of risk behaviors (Maharaj et al., 2009). Caribbean youth often experience early sexual initiation and high levels of risk factors such as violence and substance use in their environments (Pilgrim & Blum, 2012). Specifically in The Bahamas, the HIV prevalence has been historically high with new diagnoses often observed among middle-to-late adolescents (Programme NHA).

**Figure 1.**
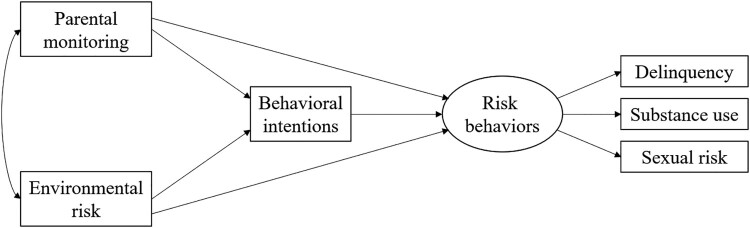
Hypothesized model of social environmental factors and adolescent risk behaviors.

As part of a nationwide implementation study of a school-based, evidence-based HIV prevention program in The Bahamas (*Focus on Youth in the Caribbean* (FOYC) and *Caribbean Informed Parents and Children Together* (CImPACT)), Grade 6 students from 24 government primary schools completed a pre-implementation assessment in 2019. These data allowed us to model youth risk behaviors along with their perceptions of parental monitoring, environmental risk, and behavioral intentions prior to receiving the HIV prevention program. Based on the results of prior research and the social-ecological model, we constructed a hypothesized model (Figure 1) in which behavioral intentions mediate the effects of parental monitoring and environmental risk on adolescent risk involvement. In addition, parental monitoring and environmental risk were hypothesized to have direct effects on risk behaviors. This analysis tests a model of the dynamics of social environmental factors influencing adolescent risk behaviors.

## Method

### Participants and study site

Participants were 2205 Grade 6 students (1091 males and 1114 females) from 24 government elementary schools in New Providence, The Bahamas. The mean age of the students was 10.5 years (range 9–13 years, with 93% of the sample being 10 or 11 years old). Approximately 99% of participants were of African descent. These cross-sectional data came from the students’ baseline (i.e. pre-assessment) survey in 2019 from a hybrid type 3 implementation evaluation of FOYC + CImPACT. Parents and students were informed that participation was voluntary, and students’ answers were confidential. Written youth assent and parent consent were required for participation. The research protocol was approved by the UMass Chan Medical School Institutional Review Board and the Institutional Review Board of the Bahamian Princess Margaret Hospital, Public Hospitals Authority.

### Procedure

Data were collected in students’ classrooms with an anonymous pencil-and-paper questionnaire, the Bahamian Youth Health Risk Behavioral Inventory (BYHRBI) (Deveaux et al., 2007), which included all subscales described below. The questionnaire was read out loud by project staff while the students marked their responses on the questionnaires, which required approximately 45 minutes to complete. Teachers left the classroom during the survey.

### Instrumentation

#### Parental monitoring

Parental monitoring was measured using an eight-item scale that assessed students’ perceptions of their parents’ monitoring efforts on a 5-point Likert scale (1 = never to 5 = always) (Li et al., 2000). Adolescents rated parental monitoring in three domains: parental knowledge of their whereabouts (two items: e.g. ‘Before I go out, I tell my parents who I am going to be with.’), youth disclosure about their activities (three items; e.g. ‘I talk to my parents about things that happen in school.’), and parental supervision (three items; e.g. ‘When I go out, I come back home at the time my parents/guardians say that I should.’). The average scale score ranged from 1 (low level of parental monitoring) to 5 (high level of parental monitoring). The Cronbach alpha for the internal consistency of the entire scale was 0.75.

#### Environmental risk factors

Environmental risk factors were measured with a 12-item scale, assessing students’ perceptions of the frequency of violence, sexual activity, alcohol consumption, and drug use in their environment. Response options were measured on a 3-point scale (1 = never to 3 = very often). Questions included ‘How often do you see people fighting?’ and ‘How often do you see people drinking [alcohol]?’ The average of the 12 items yielded a scale score ranging from 1 to 3. The internal consistency of the measurement was 0.68.

#### Behavioral intentions

Behavioral intentions were measured with a nine-item scale, assessing students’ intentions to participate in risky behaviors including substance use, sexual risks, and delinquency. Items were measured on a 5-point scale (1 = ‘No chance in the world’ to 5 = ‘Yes, big chance that I would’). Example questions included, ‘What are the chances that you could be persuaded to become pregnant or make somebody pregnant before you finish school?’ and ‘What are the chances that you would sell drugs if you were broke?’ The average of the items yielded a scale score ranging from 1 to 5. The internal consistency of the scale was 0.75.

#### Adolescent risk behaviors

A youth's risk behavior involvement in the past 6 months was assessed using three subscales (delinquency, substance use, and sexual risk behaviors). Youth were asked to report on whether they had engaged in four types of delinquent and aggressive behaviors: whether they had been truant; had carried a knife/screwdriver as a weapon; had been involved in stealing or breaking into a home, shop or business; and had engaged in a fight. The composite score for adolescent delinquency ranged from 0 to 4, with higher scores indicating more-extensive risk involvement. The questions assessing youth involvement in substance use included smoking cigarets, drinking alcohol, and using marijuana. The composite score for substance use behavior ranged from 0 to 3. Sexual risk behaviors were assessed by three questions: whether the youth had ever had sex, had sex in the past 6 months, and did not use a condom during the last sexual encounter. The sexual risk composite score ranged from 0 to 3, with higher scores indicating higher sexual risk. Following accepted practices in the literature (Bornovalova et al., 2008), the investigators assigned the same weight to each risky behavior in constructing the composite scores for the three types of risky behaviors.

### Data analysis

First, the proportions of students involved in risky behaviors were calculated for the total sample and for males and females separately, and a chi-square test was used to compare the proportions of male or female students who engaged in each risky behavior. Second, we used Spearman correlations to examine the associations between age, parental monitoring, environmental risk, behavioral intentions, delinquent behaviors, substance use, and sexual risk behaviors. Third, we conducted structural equation modeling to examine the relationships among factors influencing adolescent risk behaviors using the Mplus 7 (Muthén LKaM, 1990-2017). Model testing involved consideration of the hypothesized model (Figure 1) followed by modifications to improve model fit. In the proposed model, we tested whether parental monitoring and environmental risk had direct effects on behavioral intention, which in turn predicted adolescent risk behaviors. We also tested whether parental monitoring and environmental risk had a direct effect on adolescent risk behaviors. We included student's age and gender as covariates. Mediation effects were tested using the Sobel test, a commonly used method (Sobel, 1982). Standardized regression coefficients for all paths were estimated using maximum likelihood (ML) estimation. Missing data was handled using full information maximum likelihood (FIML). Goodness of model fit was assessed using standardized root mean square residual (SRMR), Bentler's comparative fit index (CFI) and the Tucker Lewis Index (TLI), and root mean square error of approximation (RMSEA). Good model fit corresponded to an SRMR and RMSEA less than 0.05, and values of CFI and TLI greater than 0.95 (Byrne, 2013; Hu & Bentler, 1999).

## Results

### Risky behaviors among Grade 6 students

Table 1 displays the proportion of students (total, male, and female) who participated in the 11 risk behaviors in the six months prior to completing the survey. Males participated in significantly more risk behaviors than females across all items. A total of 54.3% of the sample had engaged in a fight, the most common delinquent behavior (65.2% males vs. 43.7% females; χ^2^ = 103.03; *P* < 0.001). The most common substance used was alcohol (21.7% total; 27.8% males vs. 15.7% females; χ^2^ = 46.84; *P* < 0.001), and 7.0% of the sample had ever had sex (10.8% males vs. 3.2% females; χ^2^ = 48.44; *P* < 0.001).

**Table 1.** Proportion of adolescents involved in delinquent, substance use, and sexual risk behaviors in Grade 6.

	Total	Males	Females	χ^2^	*P*
Sample (*n*)	2205	1091	1114		
Delinquent Behaviors					
1. Was truant	14.0%	16.0%	12.0%	7.05	0.0079
2. Carried a knife/screwdriver	4.3%	6.9%	1.7%	36.36	<.0001
3. Carried a gun	2.8%	4.8%	0.7%	34.23	<.0001
4. Engaged in a fight	54.3%	65.2%	43.7%	103.03	<.0001
5. Involved in stealing/burglarizing	4.5%	7.2%	2.0%	33.62	<.0001
Substance Use Behaviors					
6. Smoked cigarets	4.0%	6.4%	1.7%	30.47	<.0001
7. Drank alcohol	21.7%	27.8%	15.7%	46.84	<.0001
8. Used Marijuana	1.7%	2.5%	0.9%	8.56	0.0034
Sexual Behaviors					
9. Ever had sex	7.0%	10.8%	3.2%	48.44	<.0001
10. Had sex in last 6 months	4.4%	7.4%	1.6%	42.78	<.0001
11. Risked pregnancy	1.8%	2.7%	0.9%	9.93	0.0016

### Bivariate associations between age, parental monitoring, environmental risks, behavioral intentions, and risk behaviors

The strength of associations between factors influencing youth behavioral intention and adolescent risk behaviors (delinquent behaviors, substance use and sexual risk) was examined using Spearman correlation coefficients (Table 2). Parental monitoring was negatively correlated to environmental risk, behavioral intentions, and three types of risk behaviors (*r* = −0.19 to −0.34, *p* < 0.001). Environmental risk was also positively correlated with behavioral intentions and risk behaviors (*r* = 0.17-0.32, *p* < 0.001). Behavioral intentions had comparatively strong correlations with the three types of risk behaviors (*r* = 0.28-0.39, *p* < 0.001). The three domains of risk behaviors were highly correlated with each other (*r* = 0.26-0.37, *p* < 0.001). In addition, student's age was negatively related to parental monitoring and positively related to environmental risk, behavioral intentions and all three types of risk behaviors.

**Table 2.** Spearman correlation coefficients between age, parental monitoring, environmental risk, behavioral intentions, and adolescent risk behaviors (*n* = 2205).

Variables	1	2	3	4	5	6	7	Mean	SD
1. Age	1.00							10.50	1.53
2. Parental monitoring	−0.09^a^	1.00						4.05	0.78
3. Environmental risk	0.07^a^	−0.27^a^	1.00					2.04	0.31
4. Behavioral intentions	0.11^a^	−0.34^a^	0.28^a^	1.00				1.58	0.64
5. Delinquent behaviors	0.18^a^	−0.34^a^	0.32^a^	0.38^a^	1.00			0.78	0.85
6. Substance use	0.10^a^	−0.24^a^	0.29^a^	0.39^a^	0.37^a^	1.00		0.27	0.54
7. Sexual risk	0.11^a^	−0.19^a^	0.17^a^	0.28^a^	0.26^a^	0.26^a^	1.00	0.13	0.45

^a^*P* < 0.001.

SD = standard deviation.

### Structural equation modeling

Estimation of the initial model (Figure 1) revealed a significant chi-square statistic and unacceptable CFI and TLI values (close to 0.90). In modifying the initial model, we included student's age in the model, adding direct paths from age to behavioral intention and risk behaviors. The overall fit of the revised model was excellent (CFI = 0.99, TLI = 0.97, RMSEA = 0.04, Chi-Square/DF = 3.34; SRMR = 0.02). The analysis revealed an R^2^ value of 0.57 for adolescent risk behaviors.

**Figure 2.**
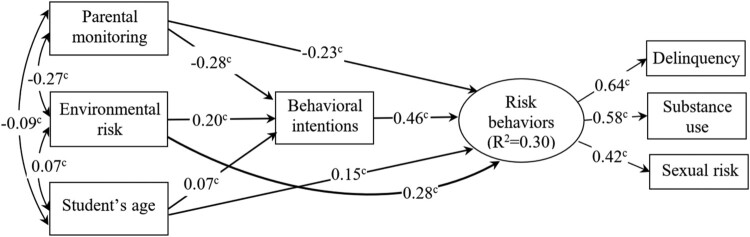
The relationships between parental monitoring, environmental risk, behavioral intentions, and adolescent risk behaviors. *R*^2^ = 0.57. ^c^*P* < 0.001.

The revised structural model demonstrated relationships among factors and their direct and indirect effects on behavioral intentions and risk behaviors (Figure 2). It had three manifest exogenous variables (parental monitoring, environmental risk, and age), one manifest endogenous variable (i.e. behavioral intentions) and one latent endogenous variable (risk behaviors) in the model. In the final model, environmental risk factors predicted behavioral intentions, which in turn predicted high levels of adolescent risk behaviors; parental monitoring negatively predicted behavioral intentions, which in turn predicted low levels of adolescent risk behaviors. Parental monitoring also demonstrated a direct negative (protective) effect and environmental risk had a positive effect on adolescent risk behaviors. Parental monitoring was negatively associated with environmental risk factors. Older age was positively related to behavioral intentions and risk-taking behaviors. The Sobel test of mediation effect indicated that behavioral intentions mediated the relationship between parental monitoring and risk behaviors (z = 11.29, *p* < 0.001) and the relationship between environmental factors and risk behaviors (z = 8.86, *p* < 0.001) and the relationship between age and adolescent risk behaviors (z = 3.44; *p* = 0.001).

## Disscussion

Our data supported our hypothesized model for the influences of parental monitoring, environmental risks, and behavioral intentions on early adolescents’ risk behaviors (delinquency, substance use, and sexual risk behaviors). Parental monitoring was a strong protective factor against behavioral intentions and engagement in risk behaviors, whereas environmental risk predicted increased behavioral intentions and risk behaviors. Behavioral intentions demonstrated a strong direct effect on adolescent risk behaviors. The entire model displayed the interconnectivity of all these factors, which is supported by the social-ecological model. This parsimonious model explained 57% of variation in risk behaviors, indicating the great influence of social environmental risk factors on adolescent behavior intentions and risk-taking behaviors. Our study has many strengths, including its large sample size of an understudied population and latent variable modeling.

### Pathways of influence

Human behavior is inherently multicausal: a multitude of environmental and social factors can support an individual's behavior. A strength of the social-ecological model is that it accounts for some of the complexity that various levels have on the development of the individual. While considering these levels, we identified direct effects of parental monitoring, environmental risk, and student age on the risk behaviors. The directions of those effects are consistent with the existing literature. For example, researchers found that students that reported high levels of parental monitoring reported fewer risky behaviors (DeVore & Ginsburg, 2005). Similarly in line with previous research, students exposed to riskier environments were more likely to participate in risk behaviors than those who were less exposed to environmental risks (Browning et al., 2015). As described by Duell et al. (2018), older students were more likely to engage in delinquency, substance use, and sexual risks than younger adolescents. This might be because older students were more physically or emotionally developed, were allotted more independence, and/or were more interested in and capable of experimenting.

The model also illuminated several indirect effects. Behavioral intentions had a mediating effect for the other measures and risk behaviors. Behavioral intentions were also strongly related to risk behaviors. Although the adolescents in this sample were young, the strong relationship between intention and engagement in risk behaviors means that behavioral intentions could be used as a proxy measure when identifying youth for a prevention intervention. It is recommended to start sex education for children around the age of 10 years old (Igras et al., 2014), in the hope of teaching them safe sex skills before they become sexually active. Thus, having a proxy measure for risky behaviors may allow researchers and practitioners to identify at-risk youth more easily. Early sex education also delays sexual initiation, which helps avoid negative health consequences associated with sex.

Parental monitoring also had a negative relationship with environmental risk. This might be because parents who provide more monitoring might be able to steer their children away from risky environments or situations. Potentially, increasing parental monitoring could be an effective mitigation strategy in risky environments.

### Gendered proportions of risky behaviors

It was interesting that males engaged in each risky behavior significantly more than females. However, both groups had the same relative rankings on prevalence of risk behaviors (i.e. engaging in a fight was the most common risk behavior for both genders, followed by drinking alcohol, and being truant from school). The discrepancy between genders may be partially explained by cultural norms and fears of teenage pregnancy driving parents to monitor their daughters to a greater extent than their sons (Dávila et al., 2017). Potentially, females organically participated in fewer risk behaviors, or they were under higher scrutiny from their parents.

### Limitations

Two limitations of this study were that data were self-report and only collected at one timepoint. The students may have been subject to social desirability and/or recall bias. Thus, they may have over- or under-reported their risky behaviors. Because data were only collected at one time, we were not able to determine whether students answered consistently across time (e.g. reporting they had sex at baseline but not at a secondary time point). Further, the cross-sectional design of this study does not allow us to infer causality. Though SEM assumes some level of prediction of the relationship between variables, causality cannot be assumed from these data.

A third limitation was that, in developing the composite score of three types of risk behaviors, we assigned the same weight to all behaviors, although arguably some behaviors may be less healthy in the short and long term. The strategy of creating a composite score of risk behaviors without differential weighting is supported by the literature (Bornovalova et al., 2008).

## Conclusions

Our findings add to previous research and our understanding of the way social environmental factors interact with one another and influence adolescent delinquency, substance use, and sexual risk behaviors. First, these results confirm that environmental risk factors increase the likelihood that early adolescents will engage in risky behaviors. Second, our results showed that behavioral intentions can act as a mediating factor between risk/protective factors and risk behaviors. Finally, the findings suggested that, for Bahamian adolescents, increased parental monitoring could be a potential mitigating factor to prevent them from engaging in risky behaviors. Several studies have produced findings that suggest the *quality* of parental monitoring is imperative to act as a protective factor against adolescent risk behaviors. For example, higher levels of family connectedness led to fewer adolescent sexual risk behaviors (Negeri, 2014), and parents who provided their children support as opposed to control saw fewer risky behaviors (Kerpelman et al., 2016). There is an opportunity to place more focus on training parents to provide quality support to lessen factors that increase adolescent risk behaviors and to enhance school-based prevention programs (Stanton et al., 2015). These combined results elucidate a potential avenue to protect youth from participating in risky behaviors.
